# Recent advances in skeletal muscle physiology

**DOI:** 10.1016/j.bjae.2023.12.003

**Published:** 2024-01-22

**Authors:** V. Kaura, P.M. Hopkins

**Affiliations:** Leeds Institute of Medical Research at St James's, University of Leeds, UK

**Keywords:** excitation–contraction coupling, malignant hyperthermia, muscle fatigue, sarcopenia, skeletal muscle

**Key points**

• Skeletal muscle accounts for up to 75% of body proteins and is important in several physiological processes.
• Excitation–contraction coupling is dependent on sarcoplasmic reticulum (SR) calcium release following key interactions between Ca_v_1.1 and type 1 ryanodine receptor (RYR1).
• Novel extracellular calcium entry pathways play a prominent role in skeletal muscle function.
• Oxidative phosphorylation is critical for skeletal muscle adenosine triphosphate synthesis.
• Cellular derangements in muscle lead to diseases such as malignant hyperthermia, critical illness myopathy, and sarcopenia.

**Learning objectives**

By reading this article, you should be able to:

• Discuss the key proteins involved in excitation–contraction coupling.
• Explain resting calcium entry, excitation-coupled calcium entry, and store-operated calcium entry.
• Describe how impairments in key skeletal muscle proteins lead to diseases managed by anaesthetists and intensivists.

Skeletal muscle is the largest mass of tissue in the body, accounting for 50%–75% of total body proteins and approximately 40% of total body weight.^1^ It plays key roles in motor function, heat generation, substrate storage, and glucose metabolism, perturbations of which leads to disease states. The basics of skeletal muscle physiology have been previously reviewed in this journal.^2^ Some of the key concepts are revisited in this review to help understand the recent advances in the field, together with their clinical importance.

## Skeletal muscle physiology

### Generation of an action potential

**Fig 1:**
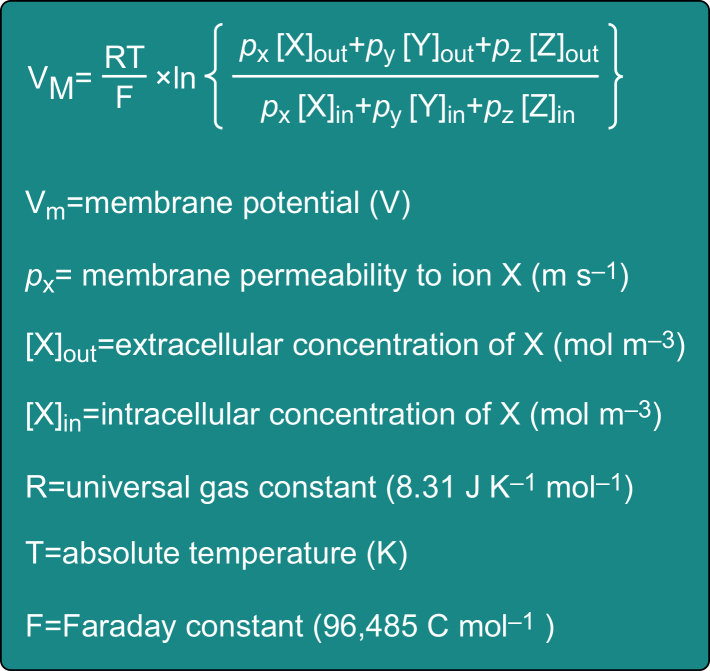
The Goldman–Hodgkin–Katz voltage equation is used to calculate the membrane potential in a multi-ion system and can help estimate the sarcolemma membrane potential. It is a modification of the Nernst equation described previously and can be used to determine the effect on the membrane potential of altering the intra- or extracellular concentrations of several different ions.^2^

Muscle fibres, like neurones, are excitable cells with a resting membrane potential of -70 to -90 mV. Their cell membrane, which is also known as the sarcolemma, contains the ion channels, pumps, and voltage-gated ion channels that are necessary to maintain this negative membrane potential and for the generation of an action potential. The resting membrane potential is a function of the net electrochemical gradients of ions that a membrane is permeable to at that time. It can be calculated using the Goldman–Hodgkin–Katz voltage equation (Fig. 1), which is a modification of the Nernst equation that incorporates multiple ions. The plasma and cytoplasmic concentrations of several ions together with the Nernst equation have been discussed previously.^2^

*In vivo*, the generation of a muscle action potential is initiated by nerve impulses from the alpha motor neurone that arrive at the neuromuscular junction and lead to the release of acetylcholine (ACh) into the synapse. The binding of ACh molecules to nicotinic ACh receptors on the post-junctional sarcolemma induces motor endplate potentials, which upon exceeding the threshold membrane potential result in the activation of Na_v_1.4 voltage-gated sodium channels. The *SCNA4* gene encodes the Na_v_1.4 channels which are highly expressed in skeletal muscle, especially at the motor endplate.^3^ Their physiological importance is highlighted by gain- or loss-of-function variants in the *SCNA4* gene affecting the channel gating properties that lead to skeletal muscle disorders such as sodium channel myotonia and congenital myopathy, respectively.^3^ Such conditions are referred to as channelopathies because a dysfunction in the ion channel leads to a disease.

**Fig 2:**
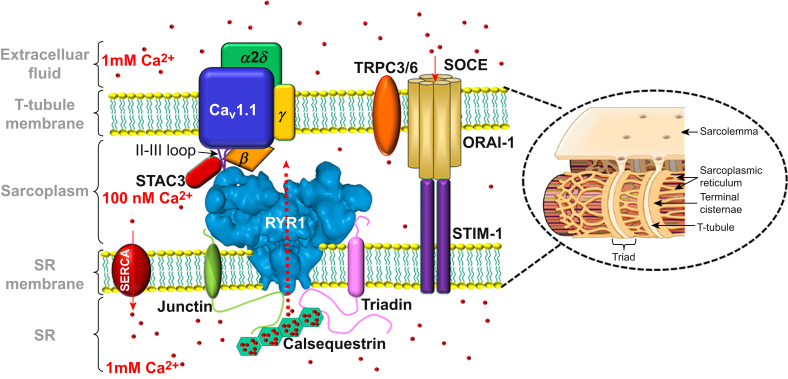
Key structures involved in excitation–contraction coupling and store-operated Ca^2+^ entry (SOCE). A change in the t-tubular membrane potential induces a conformational change in the Ca_v_1.1 subunit of the Ca_v_1.1 complex whereby the II–III loop directly interacts and activates the type 1 ryanodine receptor (RYR1); this subsequently releases sarcoplasmic reticulum (SR) Ca^2+^, thus permitting muscle contraction. The SR contains approximately 1 mM of free Ca^2+^ with further releasable Ca^2+^ stores bound within calsequestrin. Any excess cytosolic Ca^2+^ is actively pumped back into the SR by the sarco/endoplasmic reticulum Ca^2+^-ATPase (SERCA) pump. This together with RYR1 deactivation allows muscle relaxation. SH3 and cysteine-rich domain 3 (STAC3) helps chaperone Ca_v_1.1 to the sarcolemma membrane and stabilises the Ca_v_1.1–RYR1 coupling. The STIM1–ORAI interaction facilitates SOCE—other proteins such as TRPC3/6 may also contribute to SOCE, Ca^2+^ entry at rest (R_CaE_), or both. STIM1 functions as the SR Ca^2+^ sensor with ORAI forming a complex that permits extracellular Ca^2+^ into the cell. Inset: A skeletal muscle triad consists of a t-tubule sandwiched between two SR terminal cisternae; this ensures the proteins involved in EC coupling are closely apposed, thereby allowing the rapid transduction of the electrical signal into mechanical contraction (adapted from OpenStax, Skeletal Muscle, used under CC-BY-4.0). STIM1, stromal-interacting molecule 1; TRPC, transient receptor potential canonical.

The Na_v_1.4-triggered muscle action potential spreads as a wave over the sarcolemma and is rapidly propagated deep into the skeletal muscle fibres via sarcolemmal invaginations called transverse (t)-tubules (Fig. 2, inset). The t-tubule structures allow for the sarcolemma to closely appose the intracellular calcium (Ca^2+^) stores of the sarcoplasmic reticulum (SR) forming a triadic ultrastructure which consists of a t-tubule sandwiched between two terminal cisternae of the SR membrane. Junctophilins and Synaptophysin like 2 (also known as mitsugumin 29) proteins mediate the formation of the triad which provides a nanodomain for numerous protein interactions that are important for skeletal muscle function.^4^

The neuronal-mediated activation of skeletal muscle is exploited by neuromuscular monitors commonly used to identify the degree of skeletal muscle block following the use of neuromuscular blocking drugs. However, skeletal muscle action potentials can also be triggered directly using electricity (as seen during electrocution), or by chemical depolarisation using compounds such as potassium chloride. The Goldman–Hodgkin–Katz voltage equation (Fig. 1) reveals that increasing the extracellular potassium concentration leads to a membrane depolarisation. Consequently, *in vitro* and *in vivo* studies investigating skeletal muscle physiology use either potassium chloride or electrical stimulation to activate skeletal muscle tissue in their experiments.

### Excitation–contraction coupling

Excitation–contraction coupling (ECC) describes the rapid, highly organised process whereby the sarcolemmal action potential leads to the activation of SR Ca^2+^, muscle contraction followed by restoration of pre-contractile cytoplasmic Ca^2+^ concentrations. The muscle action potential generated by Na_v_1.4 is sensed by the voltage-gated calcium channel (Ca_v_1.1 complex) found within the t-tubular membrane; this then proceeds to activate the release of stored intracellular Ca^2+^. The Ca_v_1.1 complex was classically known as dihydropyridine receptors because of their amino acid sequence homology with other L-type voltage-gated Ca^2+^ channels. However, in skeletal muscle the Ca_v_1.1 complex primarily functions as a voltage sensor and not as a Ca^2+^ channel because permeability through the Ca_v_1.1 complex is not required for ECC, or for routine skeletal muscle activity.^5^ This contrasts with ECC in cardiac muscle, whereby the Ca^2+^ influx through Ca_v_1.2 (the cardiac analogue of Ca_v_1.1) is necessary to activate SR Ca^2+^ release.

The Ca_v_1.1 complex consists of a pore forming Ca_v_1.1 protein (previously known as the α1s subunit) which is encoded by the *CACNA1S* gene, plus the auxiliary subunits α2δ, β, and γ that are involved in modulating the membrane trafficking plus gating properties of the channel (Fig. 2).^6^ The t-tubule action potential induces a conformational change in Ca_v_1.1, whereby the cytoplasmic loop between the second and third transmembrane domains (II–III loop) of this channel is projected deeper into the cytoplasm, providing a physical interaction that activates the type 1 ryanodine receptor (RYR1; Fig. 2). The triadic ultrastructure allows Ca_v_1.1 complex tetrads on the sarcolemma to appose RYR1 tetramers on the SR, thus facilitating the ability of the t-tubule membrane depolarisation to rapidly activate the RYR1-mediated release of intracellular Ca^2+^ stores. This Ca_v_1.1–RYR1 coupling is bidirectional, the classical orthograde (forward) signalling is what leads to SR Ca^2+^ release. However, studies have now revealed that a retrograde signal from RYR1 affects the Ca_v_1.1 channel expression and modulates its function; the loss of this regulation is implicated in malignant hyperthermia (MH).^7^

Ca_v_1.1, RYR1 together with several other proteins, including calsequestrin, triadin, junctin, junctophilin, and mitsugumin 29, were known to form the calcium release units (CRUs) involved in physiological ECC.^4^ Recently, the SH3 and cysteine-rich domain 3 (STAC3) protein encoded by the *STAC3* gene has been discovered to also play a key role in ECC.^8^ STAC3 is specific to skeletal muscle where it helps chaperone Ca_v_1.1 to the t-tubule sarcolemma, then stabilises the coupling between the Ca_v_1.1 and RYR1 proteins through interacting with the aforementioned II–III loop of Ca_v_1.1. STAC3 is also involved in muscle differentiation and therefore is important in skeletal muscle development.^9^ The clinical importance of this protein is demonstrated by the finding that a pathogenic variant in *STAC3* results in an autosomal recessive myopathy known as STAC3 disorder which is characterised by congenital muscle weakness, delayed motor development, multiple joint plus skeletal muscle abnormalities, distinctive facies, and susceptibility to MH.^9^

In ECC, RYR1 activation permits SR Ca^2+^ release down its concentration gradient into the sarcoplasm to allow skeletal muscle contraction. In skeletal muscle the SR is the primary intracellular Ca^2+^ store that contains a total Ca^2+^ concentration of around 11 mM; the majority of this bound to calsequestrin, a Ca^2+^-binding protein, with a free SR Ca^2+^ concentration [Ca^2+^]_SR_ of approximately 1 mM available for immediate release into the cytosol. RYR1 activation leads to an increase in the basal cytosolic Ca^2+^ concentration ([Ca^2+^]_i_) from approximately 100 nM to the high nanomolar/low millimolar range. The excess Ca^2+^ is able to bind to troponin C and induce a conformational change in the troponin complex which subsequently exposes the myosin-binding sites on the actin chains within the thin filaments. The myosin heads present within the thick filaments bind and actively pull on the actin chains to cause a shortening of the sarcomere and therefore muscle contraction. This actin–myosin interaction is dependent on the presence of adenosine triphosphate (ATP) to provide the energy for sarcomeric shortening as well as sufficiently high concentrations of cytoplasmic Ca^2+^.

The released Ca^2+^ is primarily actively pumped back into the SR by the sarco/endoplasmic reticulum Ca^2+^-ATPase (SERCA) pumps to help re-establish and maintain the low basal [Ca^2+^]_i_ of approximately 100 nM. However, other pumps such as the sodium–calcium exchanger (NCX) found on the sarcolemma (and to a lesser degree mitochondria) and the plasma membrane Ca^2+^-ATPase also participate in removing excess cytosolic Ca^2+^.^10^ When the Ca^2+^ removal from the cytoplasm exceeds the SR Ca^2+^ released into it, muscle relaxation is initiated because the lower [Ca^2+^]_i_ facilitates the dissociation of Ca^2+^ from troponin C. Subsequently, the myosin heads are then unable to bind to actin; therefore, the sarcomeres passively recoil to their resting length. The need for a normal arrangement and function of the sarcomeric proteins is evident through the findings that genetic variants that affect these proteins lead to myopathies with various patterns of weakness and dysfunction.^11^ The anaesthetic management of patients with neuromuscular disorders has been described previously in this journal.^12^

### Energy utilisation

Both muscle contraction and relaxation require energy derived from ATP to be instantaneously available for rapid and repeated activity. However, the instability of ATP means that it is only available to maintain contractions for less than 1 s.^1^ Therefore, skeletal muscle uses creatine phosphate as an energy reservoir that can provide energy for a further 5–8 s of skeletal muscle activity.^1^ This is achieved by utilising the cytosolic form of creatine kinase to catalyse the release of the high-energy phosphate bond from creatine phosphate in the presence of adenosine diphosphate (ADP), to produce creatinine and ATP. Where skeletal muscle activity is more prolonged, intermediary metabolism in the mitochondria is used to generate an ongoing source of ATP by aerobic and anaerobic respiration.

Aerobic metabolism is much more efficient at generating ATP by providing 19-fold more ATP (up to 38 moles) from 1 mole of glucose, compared with only 2 moles of ATP generated by anaerobic metabolism. Nevertheless, anaerobic metabolism provides ATP that is more readily available and is independent of oxygen; the latter is critical during periods of strenuous exercise where the oxygen supply is inadequate despite maximum oxygen delivery. However, persistent high-intensity muscle activity becomes limited during anaerobic metabolism because of increasing oxygen debt and muscle fatigue. It was conventionally believed that this fatigue was a consequence of the accumulation of lactate, a strong acid that is a by-product of anaerobic metabolism, although evidence now suggests that both the acidosis and the accumulation of inorganic phosphate (P_i_) synergistically contribute to muscle fatigue.^13^

The importance of aerobic metabolism in meeting the skeletal muscle energy demands becomes apparent in mitochondrial disorders such as the metabolic myopathies which commonly display skeletal muscle phenotypes. These genetic disorders involve inborn errors of metabolism encompassing glycogen storage, fatty acid oxidation, or mitochondrial disorders, which lead to impaired ATP production.^14^ Their skeletal muscle presentation can vary from neonatal hypotonia to patients exhibiting myalgia, cramps, or rhabdomyolysis during exercise as seen in carnitine deficiency or carnitine palmitoyl transferase deficiency.^14^ A disruption in skeletal muscle utilisation of fats in intermediary metabolism may underlie the metabolic acidosis, rhabdomyolysis, and hyperthermia found in propofol infusion syndrome.^15^

In the mitochondrial myopathies, impairments in oxidative phosphorylation causes ATP deficits that result in a group of progressive muscle conditions characterised by muscle fatigue, weakness, and exercise intolerance.^14^ For example in Barth syndrome, mutations in the *TAFAZZIN* gene leads to impairments in the mitochondrial membrane protein cardiolipin. Normal functioning of cardiolipin is necessary for the electron transport chain, with abnormalities leading to a skeletal muscle myopathy, cardiomyopathy, and growth delay.^16^ The rarity of many of the mitochondrial conditions renders it difficult to provide evidence-based recommendations for the perioperative care of for each individual syndrome. Therefore, it would be wise to use the general principles of anaesthesia that have been suggested in the management of patients with mitochondrial diseases and individualise these to the patient.^17^

## Selected advances in skeletal muscle calcium physiology

Classically it was thought that in contrast to cardiac muscle, extracellular calcium entry does not play an important role in skeletal muscle physiology. Research over the last two decades suggests this is not accurate, with growing evidence supporting that extracellular Ca^2+^ entry through different mechanisms has a significant impact on skeletal muscle function. It is important to appreciate that although the skeletal muscle SR is the major intracellular store of Ca^2+^, the extracellular fluid also contains a significant readily exchangeable pool of Ca^2+^ of approximately 30 mmol, with an ionised Ca^2+^ concentration of 1 mM.

### Store-operated calcium entry

Store-operated calcium entry (SOCE) is a process that was first described in salivary gland cells and has since been found to be a key pathway for the entry of extracellular Ca^2+^ influx in non-excitable cells. SOCE provides cells with a mechanism that permits an uptake of extracellular Ca^2+^ during times when intracellular stores of Ca^2+^ are depleted.^18^^,^^19^ It classically relies on the interaction of two major proteins, stromal-interacting molecule 1 (STIM1) and ORAI calcium release-activated calcium modulator 1 (ORAI1, encoded by the *ORAI1* gene). The former is the Ca^2+^ sensor in the endo/sarcoplasmic reticulum, whereas the latter is a plasma membrane Ca^2+^ channel that consequently permits the entry of extracellular Ca^2+^ (Fig. 2).^19^ SOCE has now been shown to also play an important role in skeletal muscle, where it acts to maintain the SR Ca^2+^ fibre content both for ECC and for muscle-specific gene expression.^18^ In skeletal muscle other cationic channels such as transient receptor potential canonical (TRPC) channels are also thought to facilitate SOCE either directly or indirectly. It is also becoming evident that SOCE plays an important role in muscle development and its ability to maintain contractile force during periods of prolonged activity or high-frequency stimulation. The former is partly illustrated by a loss of ORAI1 function and consequently SOCE causing an alteration in skeletal muscle fibre composition towards a predominance of type 1 fibres (slower oxidative fibres with a long twitch).^19^ Interestingly, the activation of SOCE in skeletal muscle is much more rapid than that seen in other cell types, which is consistent with the need to quickly replenish intracellular Ca^2+^ to maintain the rapid repetitive activity seen in faster fibre types (e.g. type 2A and type 2X). Mouse models have shown that a loss of STIM1 or ORAI1 expression results in a phenotype consisting of a reduced muscle mass and early lethality.^20^

### Excitation-coupled calcium entry

Excitation-coupled calcium entry (ECCE) is a pathway for the entry of extracellular Ca^2+^ that was identified as playing an important part in sustaining the amplitude of the Ca^2+^ transients following repetitive or prolonged depolarisations of the sarcolemma.^21^ Although the exact channels involved in ECCE have not been conclusively identified, it appears to require both Ca_v_1.1 and RYR1 function; however, the former does not need to conduct extracellular Ca^2+^ for ECCE to function.^5^ The ECCE pathway is distinct from SOCE because it is independent of the SR Ca^2+^ content and only mediates a small influx of extracellular Ca^2+^ during normal physiological activity.^21^ Instead, ECCE appears to gather a more prominent role in pathological states such as MH (discussed later).

### Resting calcium entry

As mentioned previously, skeletal muscle myoplasmic [Ca^2+^]_i_ is approximately 100 nM, which at rest is a consequence of the equilibrium established between the fluxes of Ca^2+^ that are present on the sarcolemma, and across the membranes of intracellular organelles, in particular, the SR and mitochondria. RYR1 has been shown to account for more than one-half of the resting [Ca^2+^]_i_ in skeletal muscle through two mechanisms that are mediated by the bidirectional communication between Ca_v_1.1 and RYR1. The first is a direct result of a basal leak of Ca^2+^ through RYR1, whereas the second is through RYR1 potentiating a basal sarcolemmal Ca^2+^ influx called the resting calcium entry (R_CaE_). Pathogenic variants in *RYR1* enhance both these processes and thus lead to an elevated [Ca^2+^]_i_, but it is not known which channels mediate the elevated [Ca^2+^]_i_. So far, both the sarcolemmal NCX and TRPC channels have been implicated.^22^

## Mechanisms underlying skeletal muscle diseases

### Malignant hyperthermia

This hypermetabolic disorder of skeletal muscle Ca^2+^ regulation occurs in genetically susceptible individuals upon exposure to volatile anaesthetic agents and/or the depolarising muscle relaxant succinylcholine. The *RYR1* gene is the principal gene implicated in MH; however, variants in genes encoding the Ca_v_1.1 and STAC3 proteins are also known to confer susceptibility to MH.^23^ Patients reported to have an MH-associated variant or variants of unknown significance in these genes should be managed as described by Gupta and colleagues.^23^ Multiple lines of evidence suggest that in patients susceptible to MH, RYR1 is ‘leakier’ in the non-triggered skeletal muscle and results in an elevated myoplasmic Ca^2+^.^24^ The downstream effects of the increased Ca^2+^ includes an enhanced R_CaE_, increased production of both reactive oxygen species (ROS) and reactive nitrogen species, and altered mitochondrial activity.^24^ The latter includes increased mitochondrial Ca^2+^ content, elevated basal metabolism, and upregulation of fatty acid metabolism but a reduced oxidative capacity.^24^, ^25^, ^26^ Consequently, the skeletal muscle is primed for an MH reaction which is activated following exposure to the triggering anaesthetic agents. However, it is still not understood why an MH reaction is not always initiated despite exposure to a triggering anaesthetic; further research into this is ongoing.^23^

ECCE is postulated to be one contributory mechanism in the triggering of an MH reaction given myotubes containing different MH-related RYR1 variants displayed an increase in ECCE. Consistent with this, dantrolene, the treatment for MH, has also been found to inhibit ECCE. These findings have led to the hypothesis that ECCE could be a particular mechanism by which suxamethonium may potentiate the triggering of an MH reaction in susceptible individuals. Further research is required to substantiate the role of ECCE in MH and identify the channel(s) mediating this mechanism of Ca^2+^ influx.

The triggering of an MH reaction causes an uncontrolled release of SR Ca^2+^ through persistently open RYR1 channels, which in turn depletes SR Ca^2+^ stores. The SR stores are insufficient to sustain an MH reaction, thus triggering the activation of SOCE. SOCE permits the relatively large extracellular Ca^2+^ content to propagate myoplasmic Ca^2+^ accumulation; this leads to metabolic stimulation, muscle contractures, heat generation, and rhabdomyolysis that are seen during an MH reaction.

Treatments targeting these pathways have been shown to be successful in both *in vitro* and *in vivo* studies using human skeletal muscle and MH mouse models. Dantrolene is clinically used to stop an MH reaction. Although not conclusive, dantrolene is thought to primarily function by binding and stabilising the constitutively open RYR1 channels in a magnesium-dependent process; thus inhibiting the dysregulated Ca^2+^ release. The equipotent dantrolene analogue azumolene has been shown to inhibit SOCE in skeletal muscle, as has the use of an STIM1-blocking antibody.^27^ Inhibiting sarcolemmal TRPC3/6 channels with compounds such as SAR7334, or the SR RYR1 leak with tetracaine, causes a reduced R_CaE_ and attenuates the MH reaction.^24^^,^^28^

Recent investigations have revealed that the perturbed myoplasmic Ca^2+^ seen in mouse models and human MH does not only result in a predisposition to an MH reaction, but also results in altered glucose homeostasis and hyperglycaemia.^29^ Enhanced myoplasmic Ca^2+^ leads to increased phosphorylation of glycogen phosphorylase and glycogen synthase by a Ca^2+^-dependent kinase. This shifts cellular metabolism towards glycogenolysis which leads to hyperglycaemia.^29^ These novel mechanisms and drugs provide new avenues of research into future therapies for MH and hyperglycaemia.

### Intensive care unit–acquired weakness

Intensive care unit (ICU)-acquired weakness is a syndrome of generalised muscle weakness detected in critically ill patients for which there is no alternative plausible aetiology other than critical illness.^30^ ICU-acquired weakness can be subclassified into three conditions: critical illness myopathy (CIM), critical illness polyneuropathy, and critical illness neuromyopathy.^30^ ICU-acquired weakness has been previously discussed in this journal; therefore, we will focus on CIM, which is characterised by limb and respiratory muscle weakness with retained sensory function.^31^ In CIM there is a greater loss of type 2 over type 1 fibres, and the preferential loss of myosin and myosin-related proteins relative to actin; the latter is a feature that is unique to CIM compared with other acquired causes (e.g. acute polyneuropathy or pure sepsis) of muscle weakness in critically ill patients.^32^ Other features observed in CIM are reduced membrane excitability with an associated disturbed ECC, increased proteolysis involving the ubiquitin proteasome, calpain and caspase pathways, and altered cellular autophagy (a normal housekeeping process that enzymatically degrades intracellular protein aggregates and damaged structures including organelles).^30^^,^^32^^,^^33^ These proteolysis and autophagy pathways normalise following ICU discharge; however, the muscle atrophy persists because of impaired muscle regrowth, which is thought to caused by a decrease in the capacity for either hypertrophy or muscle regeneration.^34^ A rat CIM model revealed that the poor ECC was attributable to a reduction in the expression of RYR1, a disruption in the distribution of both sodium channels and Ca_v_1.1 in the sarcolemma, and direct effects of pro-inflammatory cytokines such as interleukin-1α on RYR1 Ca^2+^ release. Mitochondria have also been implicated in the aetiology of CIM. Their ultrastructure and metabolism are disrupted in CIM which then leads to increased ROS production and cellular ATP depletion.^30^^,^^33^ Overall, the molecular mechanisms underlying CIM are still incompletely understood. Thus, this necessitates further research to advance the development of therapies that prevent this myopathy.

### Skeletal muscle fatigue

Muscle fatigue is a reversible progressive decline in performance following activity. Increased muscle fatigue is seen in patients with exacerbated chronic inflammatory conditions, critical care patients, and in sarcopenia.^34^

The pathogenesis of muscle fatigue is complex involving either reductions in the supply of energy or the failure of the neuromuscular unit. The former is illustrated by the rapid onset of fatigue seen in patients with myophosphorylase deficiency who are unable to catabolise glycogen. With respect to the neuromuscular unit, evidence suggests that muscle fatigue can be caused by a failure to transmit action potentials, the inability of the t-tubule to spread the excitation wave, dysregulations in the Ca_v_1.1 voltage sensing, decreased RYR1 Ca^2+^ release, decreased SR Ca^2+^ reuptake, increased P_i_, and modifications in the Ca^2+^ handling and sensitivity of myofibrillar proteins.^35^ Failure of SOCE may contribute to fatigue given that an absence of SOCE in young and aged mice led to reduced force generation and increased susceptibility to fatigue during periods of intense activity.^36^

### Sarcopenia

Sarcopenia is a progressive and generalised skeletal muscle disorder that is associated with adverse outcomes ranging from increased risk of falls and fractures to impaired ability to perform activities of daily living, and increased mortality.^37^^,^^38^ This disease of ‘muscle failure’ presents with low muscle strength, low muscle quantity or quality, and if severe, low physical performance.^37^, ^38^, ^39^ Sarcopenia occurs with normal ageing but is not limited to aged individuals, as it can develop earlier because of inactivity, or secondary to chronic illnesses including cardiovascular disease.^39^ It differs from malnutrition, which is characterised by an imbalance between energy intake and expenditure, and the quality of nutrient intake.^38^ In addition, it is distinct from cachexia, which features a more pronounced inflammatory component and relies on the loss of weight, muscle, and fat tissue for diagnosis.^38^^,^^40^ Both sarcopenia and cachexia can be present in patients with cancer where they lead to a poorer physical performance and prognosis.^40^ The prevalence of sarcopenia in critically ill patients is reported to be 43%, with the patients having an associated increased mortality, longer duration of mechanical ventilation, length of ICU plus hospital stay following mechanical ventilation.^41^ However, a problem with such critical care studies is that sarcopenia is commonly diagnosed based on radiological investigations only; this ignores the key functional criteria in the diagnosis.

The pathophysiology of sarcopenia is complex, multifactorial, and incompletely understood. The fundamental cause is believed to be an imbalance between anabolic and catabolic muscle homeostasis in the presence/absence of neuronal degeneration.^38^^,^^39^ There is increased muscle fat accumulation, but a decline in the size and number of myofibres particularly type II fibres, together with reduced muscle repair and regeneration because of skeletal muscle stem cell senescence.^38^^,^^39^ Although muscle atrophy contributes to sarcopenia, the decline in muscle strength and increased susceptibility to fatigue precede the development of atrophy. Both increased oxidative stress from muscle ROS overproduction and dysregulations in Ca^2+^ handling are thought to contribute to the functional impairments.^42^ An age-dependent decline in intrinsic muscle force production in human skeletal muscle fibres was shown to involve marked reduction in Ca_v_1.1 subunit expression, together with a functional uncoupling of the Ca_v_1.1–RYR1 interaction, resulting in reduced SR Ca^2+^ release.^42^ Both human and mice studies have shown ageing results in corresponding reductions in CRUs, mitochondria, and CRU–mitochondrial pairs, and these parallel declines in SR Ca^2+^ release and increased oxidative stress.^42^ Elevated oxidative stress causes mitochondrial dysfunction which promotes further ROS production. This then perturbs several intracellular signalling pathways (including modifications of RYR1) that lead to reduced muscle mass and strength.^42^^,^^43^ These changes could be prevented in aged mice by long-term exercise.^42^

Furthermore, there is a decrease in autophagy leading to the persistence of damaged proteins and dysfunctional mitochondria, setting off a detrimental cycle that ultimately leads to the loss of myocytes.^43^ The failure in muscle anabolism is primarily a result of increased resistance to hormones such as insulin and testosterone which are potent activators of muscle protein synthesis.^43^

Treatment strategies for sarcopenia and cancer cachexia have similar goals of (i) increasing muscle mass, (ii) improving muscle and overall patient function, and (iii) enhancing physical performance.^40^ Exercise and physical activity can attenuate inflammation, activate anabolic pathways, and promote favourable metabolic adaptations.^40^ There is compelling evidence for the benefits of resistance exercise in improving skeletal muscle mass and strength, with increasing evidence for its benefit together with appropriate nutrition in sarcopenia, but such evidence is still lacking in cachexia secondary to cancer.^38^, ^39^, ^40^ Exercise-based pre-habilitation strategies have shown promise in improving aerobic fitness, although their impact on key clinical outcomes remains uncertain, with evidence being of generally poor quality.^44^ Suboptimal study design also underlies the inconsistent evidence for the benefits of exercise-based interventions following ICU discharge in survivors of critical illness.^45^ Consequently, well-designed laboratory and clinical studies into sarcopenia in the perioperative period are needed to help better inform the clinical management of patients with sarcopenia.

## Conclusions

Skeletal muscle constitutes 40% of body mass and plays a vital part in maintaining the normal human physiology. It is now becoming evident that novel pathways of extracellular calcium entry have an important role in certain skeletal muscle pathologies seen in the operating theatre and critical care. These findings provide a greater understanding of these diseases whilst revealing novel targets for future research and therapies. A contemporary knowledge of skeletal muscle physiology provides a strong foundation for clinical practice.

## Declaration of interests

PMH is the Editor-in-Chief of *BJA Open* and an editorial board member of the BJA. VK was a previous BJA editorial fellow in 2021–2022.

## MCQs

The associated MCQs (to support CME/CPD activity) will be accessible at www.bjaed.org/cme/home by subscribers to *BJA Education*.

## References

[1] Frontera W.R., Ochala J. (2015). Skeletal muscle: a brief review of structure and function. Calcif Tissue Int.

[2] Hopkins P.M. (2006). Skeletal muscle physiology. Skeletal muscle physiology. CEACCP.

[3] Cannon S.C. (2018). Sodium channelopathies of skeletal muscle. Handb Exp Pharmacol.

[4] Rebbeck R.T., Karunasekara Y., Board P.G. (2014). Skeletal muscle excitation-contraction coupling: who are the dancing partners?. Int J Biochem Cell Biol.

[5] Dayal A., Schrötter K., Pan Y. (2017). The Ca^2+^ influx through the mammalian skeletal muscle dihydropyridine receptor is irrelevant for muscle performance. Nat Commun.

[6] Zhao Y., Huang G., Wu J. (2019). Molecular basis for ligand modulation of a mammalian voltage-gated Ca^2+^ channel. Cell.

[7] Witherspoon J.W., Meilleur K.G. (2016). Review of RyR1 pathway and associated pathomechanisms. Acta Neuropathol Commun.

[8] Rufenach B., Van Petegem F. (2021). Structure and function of STAC proteins: calcium channel modulators and critical components of muscle excitation-contraction coupling. J Biol Chem.

[9] Horstick E.J., Linsley J.W., Dowling J.J. (2013). Stac3 is a component of the excitation-contraction coupling machinery and mutated in Native American myopathy. Nat Commun.

[10] Calderón J.C., Bolaños P., Caputo C. (2014). The excitation-contraction coupling mechanism in skeletal muscle. Biophys Rev.

[11] Jungbluth H., Treves S., Zorzato F. (2018). Congenital myopathies: disorders of excitation-contraction coupling and muscle contraction. Nat Rev Neurol.

[12] Marsh S., Ross N., Pittard A. (2011). Neuromuscular disorders and anaesthesia. Part 1: generic anaesthetic management. CEACCP.

[13] Sundberg C.W., Fitts R.H. (2019). Bioenergetic basis of skeletal muscle fatigue. Curr Opin Physiol.

[14] Ahmed S.T., Craven L., Russell O.M., Turnbull D.M., Vincent A.E. (2018). Diagnosis and treatment of mitochondrial myopathies. Neurotherapeutics.

[15] Hemphill S., McMenamin L., Bellamy M.C., Hopkins P.M. (2019). Propofol infusion syndrome: a structured literature review and analysis of published case reports. Br J Anaesth.

[16] Clarke S.L., Bowron A., Gonzalez I.L. (2013). Barth syndrome. Orphanet J Rare Dis.

[17] Niezgoda J., Morgan P.G. (2013). Anesthetic considerations in patients with mitochondrial defects. Paediatr Anaesth.

[18] Kurebayashi N., Ogawa Y. (2001). Depletion of Ca^2+^ in the sarcoplasmic reticulum stimulates Ca^2+^ entry into mouse skeletal muscle fibres. J Physiol.

[19] Carrell E.M., Coppola A.R., McBride H.J., Dirksen R.T. (2016). Orai1 enhances muscle endurance by promoting fatigue-resistant type I fiber content but not through acute store-operated Ca^2+^ entry. FASEB J.

[20] Li T., Finch E.A., Graham V. (2012). STIM1-Ca(2+) signalling is required for the hypertrophic growth of skeletal muscle in mice. Mol Cell Biol.

[21] Cherednichenko G., Hurne A.M., Fessenden J.D. (2004). Conformational activation of Ca^2+^ entry by depolarization of skeletal myotubes. Proc Natl Acad Sci U S A.

[22] Eltit J.M., Ding X., Pessah I.N., Allen P.D., Lopez J.R. (2013). Nonspecific sarcolemmal cation channels are critical for the pathogenesis of malignant hyperthermia. FASEB J.

[23] Gupta P.K., Bilmen J.G., Hopkins P.M. (2021). Anaesthetic management of a known or suspected malignant hyperthermia susceptible patient. BJA Educ.

[24] Lamboley C.R., Pearce L., Seng C. (2021). Ryanodine receptor leak triggers fiber Ca^2+^ redistribution to preserve force and elevate basal metabolism in skeletal muscle. Sci Adv.

[25] Chang L., Daly C., Miller D.M. (2019). Permeabilised skeletal muscle reveals mitochondrial deficiency in malignant hyperthermia-susceptible individuals. Br J Anaesth.

[26] Bojko B., Vasiljevic T., Boyaci E. (2021). Untargeted metabolomics profiling of skeletal muscle samples from malignant hyperthermia susceptible patients. Can J Anaesth.

[27] Zhao X., Weisleder N., Han X. (2006). Azumolene inhibits a component of store-operated calcium entry coupled to the skeletal muscle ryanodine receptor. J Biol Chem.

[28] Lopez J.R., Kaura V., Hopkins P. (2020). Transient receptor potential cation channels and calcium dyshomeostasis in a mouse model relevant to malignant hyperthermia. Anesthesiology.

[29] Tammineni E.R., Kraeva N., Figueroa L. (2020). Intracellular calcium leak lowers glucose storage in human muscle, promoting hyperglycemia and diabetes. Elife.

[30] Vanhorebeek I., Latronico N., Van den Berghe G. (2020). ICU-acquired weakness. Intensive Care Med.

[31] Appleton R., Kinsella J. (2012). Intensive care unit-acquired weakness. CEACCP.

[32] Friedrich O., Reid M.B., Van den Berghe G. (2015). The sick and the weak: neuropathies/myopathies in the critically ill. Physiol Rev.

[33] Batt J., Herridge M.S., Dos Santos C.C. (2019). From skeletal muscle weakness to functional outcomes following critical illness: a translational biology perspective. Thorax.

[34] Dos Santos C., Hussain S.N., Mathur S. (2016). Mechanisms of chronic muscle wasting and dysfunction after an intensive care unit stay. A pilot study. Am J Respir Crit Care Med.

[35] Allen D.G., Lamb G.D., Westerblad H. (2008). Skeletal muscle fatigue: cellular mechanisms. Physiol Rev.

[36] Wei-Lapierre L., Carrell E.M., Boncompagni S., Protasi F., Dirksen R.T. (2013). Orai1-dependent calcium entry promotes skeletal muscle growth and limits fatigue. Nat Commun.

[37] Cruz-Jentoft A.J., Bahat G., Bauer J. (2019). Writing group for the European working group on sarcopenia in older people 2 (EWGSOP2), and the extended group for EWGSOP2. Sarcopenia: revised European consensus on definition and diagnosis. Age Ageing.

[38] Cruz-Jentoft A.J., Sayer A.A. (2019). Sarcopenia. Lancet.

[39] Damluji A.A., Alfaraidhy M., AlHajri N. (2023). Sarcopenia and cardiovascular diseases. Circulation.

[40] Dunne R.F., Loh K.P., Williams G.R., Jatoi A., Mustian K.M., Mohile S.G. (2019). Cachexia and sarcopenia in older adults with cancer: a comprehensive review. Cancers (Basel).

[41] Jiang T., Lin T., Shu X. (2022). Prevalence and prognostic value of preexisting sarcopenia in patients with mechanical ventilation: a systematic review and meta-analysis. Crit Care.

[42] Michelucci A., Liang C., Protasi F., Dirksen R.T. (2021). Altered Ca^2+^ handling and oxidative stress underlie mitochondrial damage and skeletal muscle dysfunction in aging and disease. Metabolites.

[43] Csete M.E. (2021). Basic science of frailty-biological mechanisms of age-related sarcopenia. Anesth Analg.

[44] Molenaar C.J., van Rooijen S.J., Fokkenrood H.J., Roumen R.M., Janssen L., Slooter G.D. (2023). Prehabilitation versus no prehabilitation to improve functional capacity, reduce postoperative complications and improve quality of life in colorectal cancer surgery. Cochrane Database Syst Rev.

[45] Connolly B., Salisbury L., O’Neill B. (2015). Exercise rehabilitation following intensive care unit discharge for recovery from critical illness. Cochrane Database Syst Rev.

